# Amplification Target *ADRM1*: Role as an Oncogene and Therapeutic Target for Ovarian Cancer

**DOI:** 10.3390/ijms14023094

**Published:** 2013-02-01

**Authors:** Marlena S. Fejzo, Lee Anderson, Erika M. von Euw, Ondrej Kalous, Nuraly K. Avliyakulov, Michael J. Haykinson, Gottfried E. Konecny, Richard S. Finn, Dennis J. Slamon

**Affiliations:** 1 Division of Hematology-Oncology, Department of Medicine, Jonsson Comprehensive Cancer Center, University of California at Los Angeles, Los Angeles, CA 90095, USA; E-Mails: landers@ucla.edu (L.A.); evoneuw@mednet.ucla.edu (E.M.E.); okalous@hotmail.com (O.K.); gkonecny@mednet.ucla.edu (G.E.K.); rfinn@mednet.ucla.edu (R.S.F.); dslamon@mednet.ucla.edu (D.J.S.); 2 Department of Biological Chemistry, David Geffen School of Medicine, University of California Los Angeles, Los Angeles, CA 90095, USA; E-Mails: navliyakulov@mednet.ucla.edu (N.K.A.); mhaykinson@mednet.ucla.edu (M.J.H.)

**Keywords:** *ADRM1*, oncogene, ovarian cancer

## Abstract

Approximately 25,000 ovarian cancers are diagnosed in the U.S. annually, and 75% are in the advanced stage and largely incurable. There is critical need for early detection tools and novel treatments. Proteasomal ubiquitin receptor *ADRM1* is a protein that is encoded by the *ADRM1* gene. Recently, we showed that among 20q13-amplified genes in ovarian cancer, *ADRM1* overexpression was the most highly correlated with amplification and was significantly upregulated with respect to stage, recurrence, and metastasis. Its overexpression correlated significantly with shorter time to recurrence and overall survival. Array-CGH and microarray expression of ovarian cancer cell lines provided evidence consistent with primary tumor data that *ADRM1* is a 20q13 amplification target. Herein, we confirm the *ADRM1* amplicon in a second ovarian cancer cohort and define a minimally amplified region of 262 KB encompassing seven genes. Additionally, using RNAi knock-down of *ADRM1* in naturally amplified cell line OAW42 and overexpression of *ADRM1* via transfection in ES2, we show that (1) *ADRM1* overexpression increases proliferation, migration, and growth in soft agar, and (2) knock-down of *ADRM1* results in apoptosis. Proteomic analysis of cells with *ADRM1* knock-down reveals dysregulation of proteins including CDK-activating kinase assembly factor *MAT1*. Taken together, the results indicate that amplified *ADRM1* is involved in cell proliferation, migration and survival in ovarian cancer cells, supporting a role as an oncogene and novel therapeutic target for ovarian cancer.

## 1. Introduction

Ovarian cancer is the leading cause of death from gynecologic malignancy accounting for approximately 15,000 deaths in the US annually. The disease is identified primarily in its advanced stage when it is largely incurable. New approaches for detection and therapy are warranted.

Recently, we showed that out of the 214 known genes mapping to 20q13, *ADRM1*, a gene that encodes the proteasomal ubiquitin receptor *ADRM1* protein, is the most likely amplification target at 20q13 in ovarian cancer with over-expression correlating significantly with a shorter time to recurrence and death [1]. *ADRM1* encodes an integral plasma membrane protein involved in development, cell adhesion, deubiquitination, and proteolysis [2–5]. Overexpression of *ADRM1* in 293T adenovirus-transformed human embryonic kidney cells reduces degradation of short-lived proteins [5]. It has been shown that *ADRM1* binds directly to proteasome-associated deubiquitinating enzyme, UCH37, enhancing its isopeptidase activity [4,5]. Deubiquinating proteins in human cancer represent a newly emerging class of oncogenes and tumor suppressors [6]. In *ADRM1* knock-out mice, the most notable phenotype is a defect in oogenesis, suggesting that *ADRM1* normally plays a critical role in ovarian development [7]. The role *ADRM1* plays in ovarian cancer is unknown but it has been shown to be upregulated in metastatic tumor cells, and its overexpression increased the propensity of cells to engage in cell-cell interactions [2,8]. A study of commonly overexpressed genes in solid tumors by microarray analysis shows that *ADRM1* is consistently over-expressed in multiple tumor types, including lung adenocarcinoma, lung squamous cell carcinoma, bladder, colon, liver, kidney, and stomach cancer in addition to ovarian cancer [9], suggesting that this gene may be a potential target in many tumor types. Recently, it has been shown that knockdown of *ADRM1* suppresses proliferation and inhibits colorectal cancer cell migration, survival and tumorigenicity [10,11].

We have shown that amplification in ovarian cancer tumors is a major mechanism of up-regulation of *ADRM1* and that its expression increases with tumor stage and recurrence [1]. Consistent with these findings, using array-CGH and RNA expression analysis of ovarian cancer cell lines, we showed the amplification and overexpression of *ADRM1* in 18% (7/39) of ovarian cancer cell lines [12]. Among these, ovarian cancer cell line OAW42 was found to be amplified and had the highest level of overexpression and was used as a model for knock-down of expression of *ADRM1* in *ADRM1*-amplified ovarian cancer. Ovarian cancer cell line ES2 was not amplified and was used for transfection leading to *in vitro* overexpression of *ADRM1*. With a new ovarian cancer cohort in addition to these two ovarian cancer cell line models, herein we provide additional evidence that *ADRM1* plays a role in ovarian cancer.

## 2. Results and Discussion

### 2.1. Confirmation of ADRM1 Amplification in a Second Ovarian Cancer Cohort

**Figure 1:**
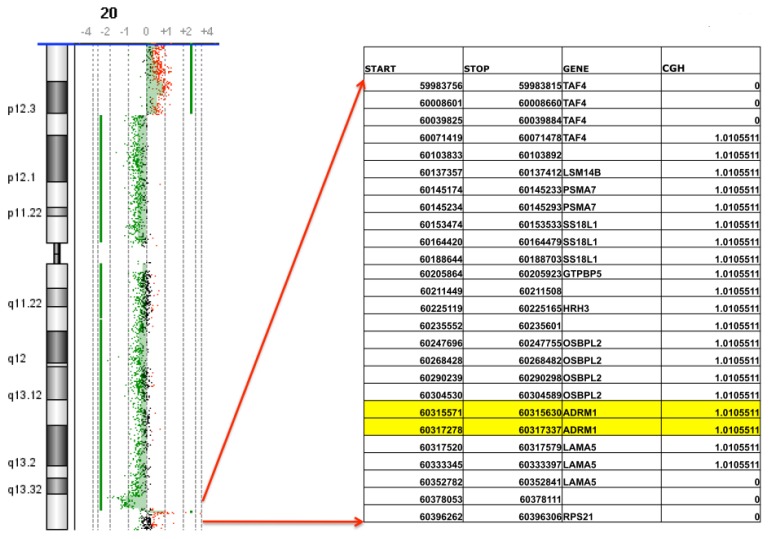
Array CGH used to define 262 KB *ADRM1* amplicon. Array CGH on DNA isolated from ovarian endometrioid stage III, grade 4 tumor reveals a 1.01-fold amplification of seven genes including *ADRM1* on 20q13.

Array-CGH analysis of 68 primary ovarian carcinomas shows DNA amplification (CGH > 0.7X) of *ADRM1* in 15% of tumors. Among the amplified tumors was a stage 3, grade 4 Endometrioid Ovarian Carcinoma with a 262 kilobase amplification of 1.01× mapping from 60,071,478 kb within the TAF4 gene on chromosome 20q13 extending to 60,333,397 kb within the LAMA5 gene on 20q13. The amplicon includes *ADRM1* and six additional genes whose expression levels do not correlate as significantly with amplification [1]. There are no gains either proximal or distal to this region on chromosome 20q in this tumor (Figure 1).

### 2.2. ADRM1 Over-Expression Increases Cell Proliferation and Conversely, *ADRM1* Knockdown Results in Significant Growth Inhibition

**Figure 2:**
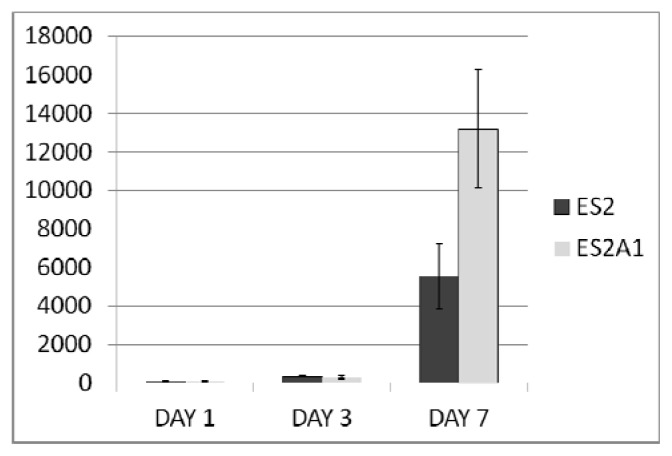
Cell counts (average of triplicate on 12-well) show a 2.2-fold increase in cell growth on day 7 in *ADRM1*-overexpressing clone ES2A1 compared to parental cell line ES2.

**Figure 3:**
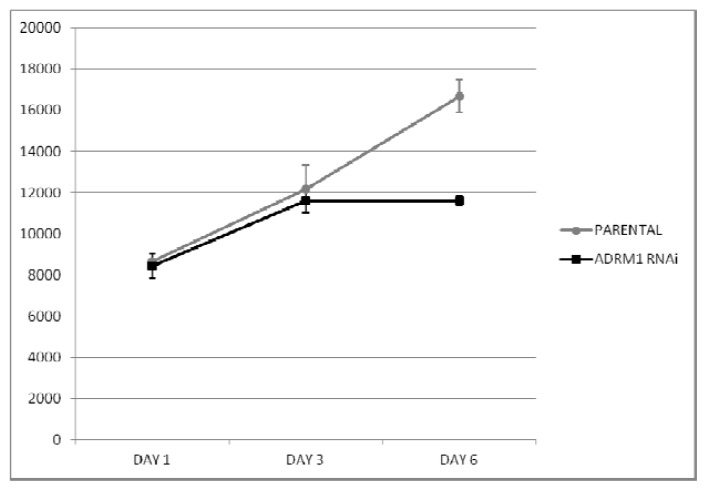
RNAi interference of *ADRM1* in OAW42 results in 1.4-fold growth inhibition compared to growth of PARENTAL (untreated) OAW42 cells.

We speculated that the preferential amplification of *ADRM1* gives overexpressing ovarian cancer cells a proliferative advantage. To determine whether or not this was the case, an ovarian cancer cell line that over-expresses *ADRM1* (ES2A1) was created by stable transfection of an *ADRM1* plasmid into ES2 cells. Cell counts in triplicate showed at least a 2.2-fold increase in cell growth on day 7 in ES2A1 cells compared to parental ES2 cells (Figure 2). In addition, naturally occurring *ADRM1*-amplified OAW42 cells treated with RNAi knock-down of *ADRM1*, resulted in a 1.4-fold growth inhibition compared to both parental *ADRM1* untreated and non-targeting RNAi treated OAW42 cells by day 7 after transfection (Figure 3). Changes in cell cycle were not detected in the overexpressing and the knock-down cell line models.

### 2.3. ADRM1 Over-Expression Increases Growth in Soft-Agar and Conversely, Knockdown of *ADRM1* Decreases Growth in Soft Agar

**Figure 4:**
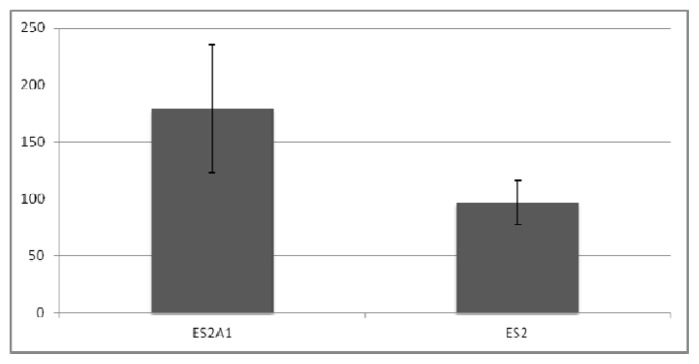
*ADRM1*-overexpressing cells are 1.9-fold more efficient in colony-formation (colony number) in soft agar than parental ES2 cells, and produce larger colonies.

**Figure 5:**
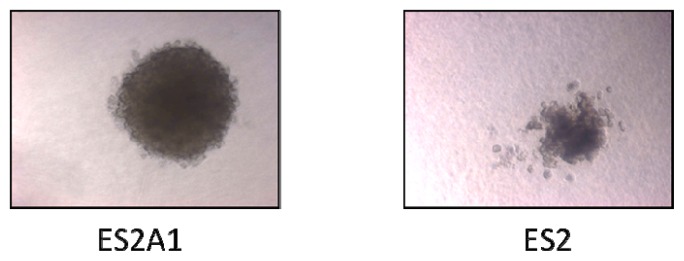
Largest ES2 colony in soft agar compared to typical ES2A1 colony in soft agar.

**Figure 6:**
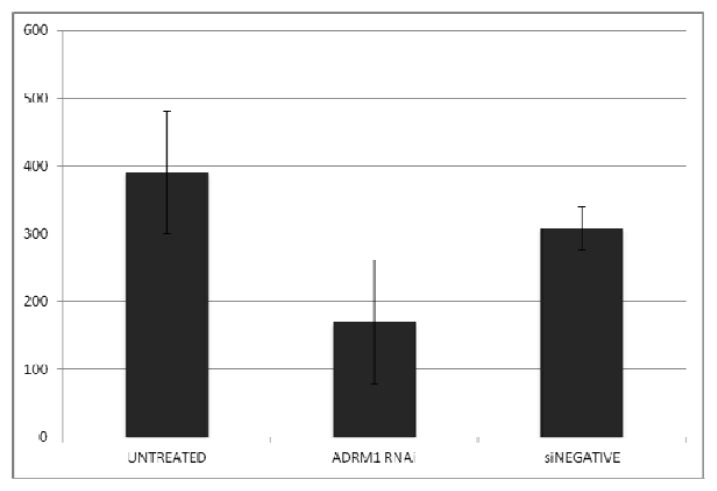
Growth in soft agar (number of colonies) in parental (untreated) compared to *ADRM1* siRNA-treated and non-targeting siRNA-treated OAW42 shows knock-down of *ADRM1* inhibits colony formation.

*ADRM1* overexpression correlates with recurrence and metastasis, and therefore, we hypothesized that expression levels effect the malignant tendency of the cells. In soft agar experiments, colony-forming efficiency in *ADRM1*-overexpressing ES2A1 cells was 1.9-fold higher than parental ES2 cells (Figure 4), and colonies are larger (Figure 5). Conversely, parental OAW42 cells showed a 2.3-fold increase and non-targeting RNAi-treated OAW42 cells showed a 1.8-fold increase in colony-formation efficiency, when compared to *ADRM1* RNAi-treated OAW42 cells (Figure 6).

### 2.4. *ADRM1* Knockdown Results in a Decrease in Proliferation and/or Migration Using the Scratch Test

**Figure 7:**
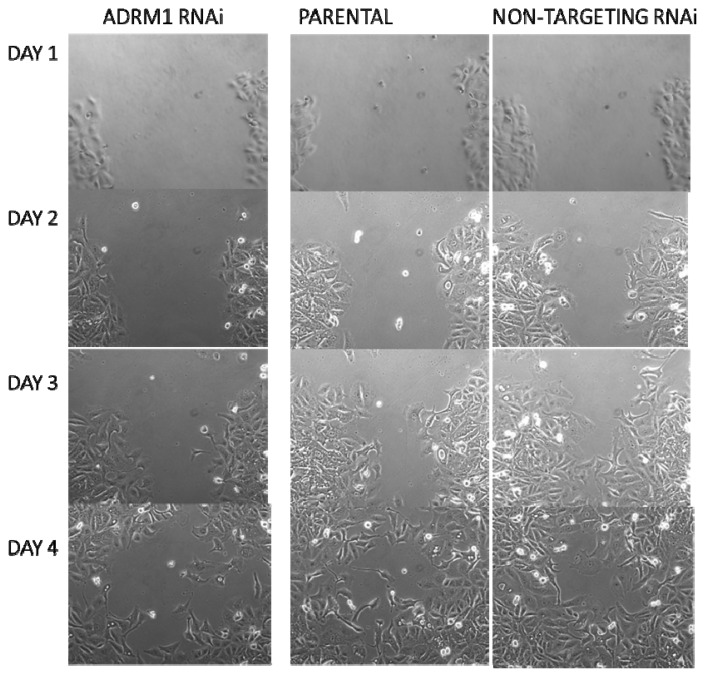
Wound-healing assay using the scratch test shows decrease in proliferation and/or migration in *ADRM1*-silenced OAW42 cells compared to non-targeting-RNAi treated OAW42 cells and parental untreated OAW42 cells.

Additionally, a wound-healing assay was performed on *ADRM1* knock-down OAW42 cells compared to both parental and non-targeting RNAi-treated OAW42 cells. A similar-sized wound was detected on the first day for each cell type. By day 3, the *ADRM1*-knock-down cells were only 50% healed, compared to 75% closing of the scratch wound in the parental and 88% in the non-targeting control cells (Figure 7).

### 2.5. Knockdown of *ADRM1* Increases Apoptosis in Ovarian Cells

**Figure 8:**
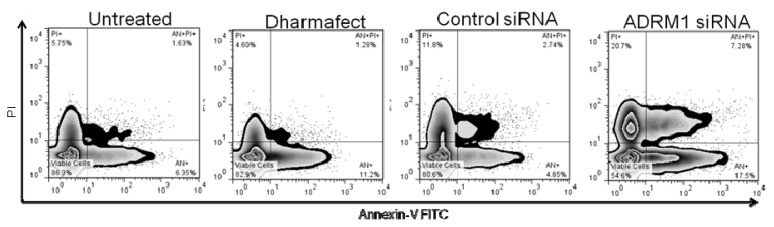
Effects on apoptosis of silencing *ADRM1* in OAW42 ovarian cells. *ADRM1* siRNA cells shows an increase in the percentage of apoptotic cells (25% Annexin-V positive) and dead cells (21% PI+) cells when compared with untreated control (8% Annexin-V positive/6% PI+), Dharmafect (13% Annexin-V positive/5% PI+) or scramble control siRNA (8% Annexin-V positive/12% PI+). No significant apoptosis or death was seen in Dharmafect or control siRNA compared to untreated OAW42 cells.

Cell cycle and apoptosis experiments were performed to determine whether *ADRM1* expression has an effect on cell cycle and whether blocking *ADRM1* results in apoptosis. Cell cycle and apoptosis were examined by flow cytometry for *ADRM1* over expressing clone ES2A1 and parental ES2, and no significant differences were detected (data not shown). Additionally, no significant differences in the cell cycle were detected between OAW42 cells with knock down of *ADRM1* compared to parental untreated OAW42, nor dharmafect-treated OAW42 (nor OAW42 treated with either of two non-specific RNAi controls) (data not shown). However, there was a 20% increase in apoptosis, both early and late apoptosis (AN+/AN+PI+), and a 10% increase in cell death (PI+ cells) in *ADRM1* RNAi-treated OAW42 cells compared to parental untreated OAW42, nor dharmafect-treated OAW42 and non-specific RNAi controls (Figure 8).

### 2.6. *ADRM1* Knockdown Results in the Down-Regulation of 15 Proteins Including MNAT1 and the Up-Regulation of Seven Proteins

**Figure 9:**
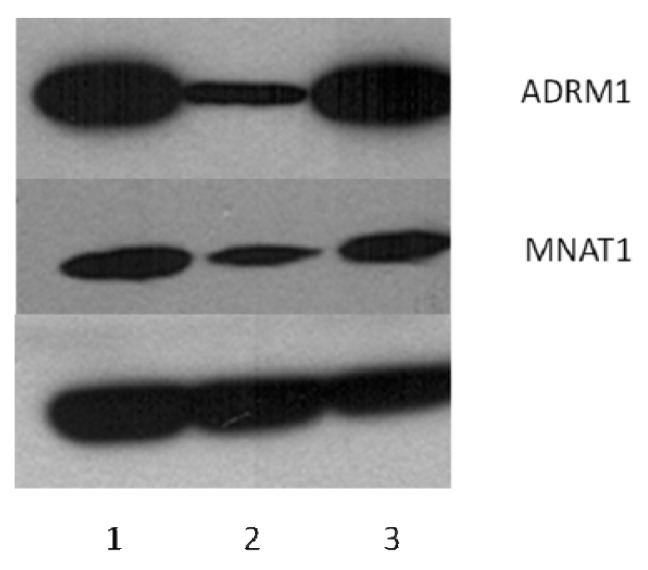
Western Blotting confirms that gene-silencing of *ADRM1* results in dysregulation of *MNAT1* (and *HAX1* and *ST1A3*, not shown). (**1**) Dharmafect-treated OAW42; (**2**) *ADRM1* RNAi-treated OAW42; (**3**) siRNA non-targeting treated OAW42.

*ADRM1* is a proteasomal ubiquitin receptor and therefore we predicted knock-down of *ADRM1* might affect the regulation of other proteins. Therefore, we performed Difference Gel Electrophoresis (DiGE) analysis on OAW42 cells with RNAi knock-down of *ADRM1* and compared protein levels to parental untreated and non-targeting RNAi-treated OAW42 cells. We identified 15 proteins that were down-regulated and 7 proteins that were upregulated when *ADRM1* is knocked-down (Table 1). These proteins were excised from the gel and identified with mass spectrometry. Three of the proteins were confirmed to be differentially expressed by Western Blot Analysis on the original lysates, including *MNAT1*, *HAX1*, and *ST1A3* (Figure 9).

### 2.7. Discussion

Recently, we showed that among 225 ovarian samples, *ADRM1* is a likely 20q13-amplification target in ovarian cancer. It was amplified and overexpressed in 23% of tumors, was significantly upregulated with respect to stage, recurrence and metastasis, and its overexpression correlates significantly with shorter time to recurrence and overall survival [1]. Consistent with these findings, using array-CGH and RNA expression analysis of ovarian cancer cell lines, we showed the amplification and overexpression of *ADRM1* in 18% (7/39) of ovarian cancer cell lines [12]. Herein, CGH analysis of *ADRM1* in a separate cohort of 68 primary ovarian carcinomas, including one with high level amplification of a 262 kilobase region containing *ADRM1*, provide additional evidence that *ADRM1* is the driver of a 20q13 amplicon in ovarian cancer.

The role of this gene was explored using *ADRM1*-upregulated and *ADRM1* down-regulated *in vitro* models. Evidence suggests that *ADRM1* has traits of an oncogene as amplification leads to increased cell proliferation and increased colony-formation efficiency. Conversely, down-regulation of *ADRM1* at the RNA and protein levels results in growth inhibition, decreased colony-formation efficiency, slower wound-healing, decreased cell viability, and significantly increased apoptosis. Knock-down of *ADRM1* resulted in the dysregulation of multiple proteins already known to play a role in cancer. In particular, multiple *HSP90* fragments were identified. A recent study showed *HSP90* inhibition leads to simultaneous inactivation of multi-RTKs including EGFR, ERBB2, MET, and AXL and suppresses the downstream survival/proliferation signaling in multiple ovarian cancer cell lines [13] making it a promising target for ovarian cancer therapy. Drugs targeting heat shock proteins are currently of intense focus, and are involved in several clinical trials in ovarian and other cancers [14].

SULT1A1 (*ST1A3*) is up regulated with the knock-down of *ADRM1* and encodes a protein that catalyzes the sulfate conjugation of many hormones. Variants in *ST1A3* are linked to increased hormone-dependant ovarian cancer risk [15,16].

The gene *HAX1* was shown in this study to be down regulated with *ADRM1* and is known to associate with hematopoeitc cell-specific Lyn substrate 1, a substrate of the Src family of tyrosine kinases [17]. *HAX1* promotes cell survival via inhibition of CASP9, responsible for apoptosis execution [18]. Therefore its down regulation may explain the significant increase in apoptosis in *ADRM1* RNAi-treated ovarian cancer in this study. It also potentiates GNA13-mediated cell migration and invasion [19,20], and thus its down regulation may contribute to the decrease in wound healing in *ADRM1* knock-down cells shown in this study. Thus one can hypothesize that the correlation between *ADRM1* over expression and recurrence and metastasis shown previously in ovarian cancer may be through regulation of *HAX1*.

Finally, the most significantly dysregulated gene, *MNAT1* (MAT1), stabilizes the cyclin H-CDK7 complex to form a functional CDK-activating kinase (CAK) enzymatic complex, which in turn activates the cyclin-associated kinases CDK1, CDK2, CDK4 and CDK6 by threonine phosphorylation [21]. Interestingly, in a mouse model for pancreatic cancer lymphatic metastasis, *ADRM1* was one of 30 genes found to be upregulated in the metastatic cell line compared to the parental cell line [22]. In another study, direct RNAi silencing of *MNAT1* suppressed the growth of pancreatic cancer cells *in vitro*, and significantly achieved an anti-tumor effect on the subcutaneously transplanted pancreatic tumor *in vivo* [23]. Thus, down regulation of *MNAT1* in *ADRM1*-silenced ovarian cancer may explain the similar anti-tumor effects shown in this study. Additionally, *MNAT1* has been shown to interact with Cyclin H, Estrogen receptor alpha, MTA1, and cyclin-dependent kinase 7, all linked previously to ovarian cancer [24–26].

## 3. Experimental Section

### 3.1. Tissue

Sixty-eight primary ovarian carcinomas were snap frozen in liquid nitrogen and prepared for DNA and RNA processing as described below. The samples consisted of 38 ovarian endometrioid carcinomas (19 stage I and II, 19 stage III and IV), 16 ovarian clear cell carcinomas (5 stage I and II, 11 stage III and IV), 11 mucinous ovarian tumors (10 stage I, 1 stage III), and 3 mixed ovarian epithelial carcinomas (1 stage I, 2 stage III and IV).

### 3.2. Cell Lines

The cell line OAW42 was obtained from the European Collection of Cell Cultures (Salisbury, UK). The cell line ES2 was obtained from the American Type Culture Collection (ATCC, Rockville, MD, USA). OAW42 and cells was cultured in DMEM (ATCC) plus 10% FBS. ES2 was cultured in McCoy’S 5A medium (ATCC) plus 10% FBS.

### 3.3. Array CGH

Genomic DNA was extracted from frozen cell pellets using the DNeasy Blood and Tissue Kit (Qiagen) with the following modifications. Seventy percent ethanol was substituted for Buffer AW2 in the final wash, and DNA was eluted in 50 mL of sterile water (Invitrogen, Carlsbad, CA, USA). The concentration and quality of the DNA were measured with the NanoDrop Spectrophotometer (NanoDrop Technologies, Wilmington, DE, USA) and by electrophoresis in 1% agarose.

Labeling and hybridization of Agilent 105K oligonucleotide CGH arrays was performed according to the manufacturer’s protocol for Human Genome CGH 105A Oligo Microarray Kit (version 5.0; Agilent Technologies, Santa Clara, CA, USA, 2008). Labeled tumor and reference DNAs were combined, annealed with COT-1 DNA (Invitrogen, Carlsbad, CA, USA) and 10× Blocking Agent (Agilent, Santa Clara, CA, USA) for 30 min at 37 °C after boiling, then hybridized to Agilent 105A arrays for 40 h at 65 °C according to the manufacturer’s instructions. After hybridization, arrays were washed according to Procedure B (which includes an ozone blocking wash), and scanned using an Agilent Scanner (G2565BA). Files were extracted using Agilent Feature Extraction software (version 9.5; Agilent, Santa Clara, CA, USA, 2007) with the default CGH protocol. Extracted arrays with a DRL Spread< 0.3 were included in the analysis.

CGH Analytics software (version 4.0; Agilent Technologies, Santa Clara, CA, USA, 2008) was used for copy number analysis, employing the ADM1 algorithm (Threshold 5), with Fuzzy Zero and Centralization corrections to minimize background noise. All map positions were based on the March 2006 NCBI 36/hg18 genome assembly. A minimum of 3 consecutive probes was required to define a region as amplified or deleted. The data was also filtered by requiring a minimum absolute average log2 ratio of 0.4. All data was inspected visually using the interactive view. Log2 ratios >0.4 (1.3-fold) were considered gain and log2 ratios >1 (2-fold) were considered amplified.

### 3.4. Analyses of Cell Proliferation

ES2 and ES2A1 cells were counted and seeded in triplicate on 12-well plates and were harvested by trypsinization on days 1, 3, and 7 and counted using a particle counter (Z1; Beckman Coulter, Inc., Brea, CA, USA). OAW42 cells were counted and seeded in triplicate on 6-well plates and treated with dharmafect only, dharmafect and *ADRM1* siRNA, or dharmafect and si-negative control as explained in the RNA interference section. Cells were harvested by trypsinization on days 1, 2, 3, and 6 after transfection and counted using a particle counter (Z1; Beckman Coulter, Inc., Brea, CA, USA). Standard deviations were calculated from triplicate experiments and shown in bar graphs.

### 3.5. *In Vitro* Apoptosis and Cell Cycle Assays

The effects of silencing *ADRM1* on apoptosis and cell cycle were evaluated using Annexin V-FITC and Propidium Iodide (PI) staining and DAPI DNA staining respectively. Cells were plated evenly and allowed to grow to log phase before being transfected with siRNA to knockdown *ADRM1* or control. Also, a vehicle for transfection Dharmafect was used as a control. For apoptosis analysis supernatant was collected, cells were washed with PBS, and trypsin was applied to release cells, which were then centrifuged at 2000 rpm for 5 min. Supernatant was aspirated and cells were then suspended in 200 and allowed to grow to log ained with 10 μL of Annexin V-FITC and 5 min. Supernatant was aspirated and cell (Medical & Biological Laboratories, Co., Woburn, MA, USA). The effects of siRNA *ADRM1* on the cell cycle were assessed using Nim-DAPI staining (NPE Systems, Pembroke Pines, FL, USA). Flow cytometry was performed using a CellQuanta (Beckman Coulter, Brea, CA, USA) flow machine, and the results were analyzed using Flow Jo software (version 7.6.5; Tree Star Inc., Ashland, OR, USA, 2011).

### 3.6. Soft Agar Assay for Colony Formation

ES2, ES2A1, OAW42, and siRNA-treated (*ADRM1* siRNA and non-targeting control siRNA) OAW42 cells were counted and seeded in triplicate to 10,000 cells per well in a 24-well culture dish containing 0.4% low-melting agarose over a 0.6% agarose layer, both in culture medium, and incubated for 3–5 weeks at 37 °C. Colonies were stained with 10% Neutral Red (Fisher Scientific, Waltham, MA, USA) and counted. Standard deviations were calculated from triplicate experiments and shown in bar graphs. Experiment was repeated twice to confirm findings. Knock-down of *ADRM1* in cells from siRNA experiments was confirmed by Western Blot with protein lysates isolated seven days after seeding.

### 3.7. Antibodies and Western Blotting

Transfected and untransfected cells were isolated two days after transfection. Cells were washed in PBS and lysed at 4 °C in lysis buffer. Insoluble material was cleared by centrifugation at 10,000× *g* for 10 min. Protein was quantitated using BCA (Pierce Biochemicals, Rockford, IL, USA), resolved by SDS-PAGE, and transferred to nitrocellulose membranes (Invitrogen, Carlsbad, CA, USA). *ADRM1*, *MNAT1*, and *ST1A3* were detected, respectively with antibodies to *ADRM1* monoclonal 3C6, *MNAT1* anti-MNAT antibody produced in rabbit, *ST1A3* anti-SULT1A1 monoclonal 1F8 (Sigma-Aldrich, St. Louis, MO, USA). Monoclonal Anti-*HAX1* antibody produced in mouse was purchased from BD Biosciences, San Jose, CA, USA. Detection was performed using chemifluorescent reagent (SuperSignal West Pico Chemiluminescent Substrate, Thermo Scientific, Rockford, IL, USA). Antialpha-tubulin mouse antibody (Calbiochem, San Diego, CA, USA) 0.1 μg/mL was used as a loading control.

### 3.8. RNA Interference

Four interfering RNAs specific for *ADRM1* were purchased from Dharmacon (ThermoFisher Scientific, Lafayette, CO, USA) as a Smartpool in addition to two non-targeting siRNA oligos to use as a controls. Transfection was performed using 0.4% Dharmafect transfection reagent and 10 nM siRNA according to the manufacturer (Dharmacon, Lafayette, CO, USA). OAW42 cells were seeded and transfected at 60% confluency in antibiotic-free media (DMEM (Cellgro, Lawrence KS, USA) supplemented with 1% l-glutamine, 0.91 μg/mL bovine insulin, 4.5 g/1 L glucose, and 3.7 g/L sodium bicarbonate). After 24 h at 37 °C, growth media with 10% fetal bovine serum and antibiotic was added. Knock-down of ADRM1 was confirmed by Western Blot at day 2, 3, 4, and 7 after transfection.

### 3.9. Plasmid Constructs and Transfection

The *ADRM1* Ultimate Human ORF Clone from IOH13967 was purchased from Invitrogen (Carlsbad, CA, USA) and cloning and transfection were performed following Invitrogen’s protocol for transfection of a mammalian expression clone. Kanamycin resistant IOH13967 clones were isolated. The plasmid DNA was subcloned into amp-resistant pcDNA 6.2/V5-DEST Mammalian expression vector. True expression clone was confirmed by testing ampicillin resistance and chloramphenicol sensitivity. Pure DNA was isolated with the Purelink HQ kit and the clone was sequenced to confirm it was in frame. Ovarian cell line ES2 was tested for Blasticidin sensitivity and a concentration of 0.015 mg/mL Blasticidin kills all ES2 parental cells. ES2 was transfected with the *ADRM1* expression vector with lipofectamine. Blasticidin-resistant clones grown in 0.015 mg/mL Blasticidin were tested for increased *ADRM1* expression by Western blot. The highest *ADRM1* expressing clone ES2A1 was used in experiments in this study.

### 3.10. Scratch Test

For the scratch assay, OAW42 cells treated with *ADRM1*-specific RNAi, non-targeting RNAi, and Dharmafect only (parental), were grown in complete growth medium until 90%–100% confluency was reached. A 3 mm cross-wound was introduced across the diameter of each plate. Cell migration was observed by microscopy and photographed at 24 h, 48 h, 72 h and 96 h later.

### 3.11. Cell Growth and Protein Extraction

OAW42 ovarian cell lines were independently grown in eighteen 100 mm regular Petri dishes in complete medium for 24 h and then each of six OAW42 ovarian cell lines were treated either with 10 nM *ADRM1*-specific RNAi or non-targeting RNAi (scrambled RNAi) and the control cells with only 0.4% of Dharmafect transfection reagent (parental) in antibiotic-free medium. After 24 h, cells were incubated in complete medium for another 24 h. Then, cells were washed two times with low-serum medium at 4 °C, followed by 2 times with PBS at 4 °C, and directly lysed in 0.3 mL of ice cold labeling buffer (7 M urea, 2 M thiourea, 4% CHAPS and 20 mM Tris-HCl, pH 8.8) and then briefly sonicated in Bioruptor (Diagenade) on ice cold water bath for 30 s on and 1 min off for a total six times at high settings. Cell lysates were centrifuged at 13,500 rpm for 15 min at 4 °C and extracted proteins were stored at −80 °C.

### 3.12. DIGE Labeling, Analysis and Mass Spectrometry

DIGE labeling, analysis and mass spectrometry were performed as described previously [27]. Briefly, protein extracts used for the dige experiments were cleaned using the methanol/chloroform method and protein pellets were resuspended in the labeling buffer and centrifuged. For the dige analysis, protein samples were labeled with the *N*-hydroxysuccinimidyl ester derivates of Cy2, 3 and 5 Dyes according to the manufactures protocol (GE Healthcare, Piscataway, NJ, USA) for 30 min on ice water in the dark and then excess Cy Dyes were quenched by adding 10 mM Lysine solution and incubated 10 min. Briefly, 50 micrograms of protein from each experimental group repeated 6 times (*ADRM1*-specific RNAi, non-targeting RNAi treated, and control cells) were labeled with the 400 pmol of Cy3 and Cy5 Dyes, respectively, to avoid any Cy Dye labeling biases. Internal standard sample was prepared by pooling equal (25 micrograms) amount of proteins from all eighteen samples and labeled with the 400 pmol of Cy2. After the labeling, 50 micrograms of Cy2, Cy3 and Cy5 labeled samples were combined with the 300 micrograms of unlabeled protein mix from the all samples and the rehydration solution was added (7 M urea, 2 M thiourea, 4% CHAPS, 1% DTT. 0.5% pH 4–7 IPG buffer, 5% glycerol, 10% isopropanol). Samples were incubated for another 20 min, briefly centrifuged for 5 min at 11,000 rpm and then applied to 24 cm pH 4–7 IPG strips (GE Healthcare), passively rehydrated overnight at room temperature. Proteins were focused according with the following steps at 200 V (hold) for 2 h, 500 V (hold) for 2 h, 1500 V (gradient) for 2 h, 8000 V (gradient) for 4 h 30 min and 8000 V (hold) for 6 h 30 min for the total of 77,000 Vh. After the IEF, IPG strips were equilibrated in a 10 mL of reducing solution (50 mM Tris-HCl, pH 8.8, 6 M urea, 30% glycerol, 2% SDS, 1% DTT, 0.002% bromophenol blue) for 15 min and then a second equilibration step was followed with 4.5% (*w*/*v*) iodoacetamide in the same solution without DTT. After reduction and alkylation of proteins, IPG strips were placed on the top of the 12.5% SDS polyacrylamide gels and sealed with the 1% agarose solution. A second dimension SDS gel electrophoresis was run in Ettan DALTtwelve system (GE healthcare) at 50 V for 3 h at 24 °C and then continued at 1 W per gel at 24 °C for overnight until bromphenol blue runs out of the gels. The next day, gels were scanned using the Typhoon Trio Variable Mode Imager (GE Healthcare) at 100 micron resolution using 488 nm laser/520BP40 filter for Cy2, 532 nm laser/580BP30 nm filter for Cy3, 633 nm laser/670BP30 for filter Cy5. Gel images were cropped using Image Quant Software (GE Healthcare, Piscataway, NJ, USA). The Decyder 2D Differential Analysis software (version 6.5; GE Healthcare: Piscataway, NJ, USA) was used for the identification of statistically significantly differentially expressed proteins. After the analysis, proteins of interests were selected based on the fold changes and student’s *t* test. Gels were fixed and then stained by Sypro-Ruby. Sypro-Ruby stained images were re-matched to the dige images and protein spots were picked from three gels using the Ettan Spot Picker (GE Healthcare) and digested with modified trypsin (Trypsin Gold, Mass Spectrometry grade, Promega, (Madison, WI, USA) using the ProGest protein Digestion Robotic Station system (Genomic Solutions, Inc, (Marlborough, MA, USA). MALDI-TOF/TOF mass spectrometry analysis was performed on an Ultraflex TOF/TOF instrument (Bruker Daltonics, Bremen, Germany) [28].

## 4. Conclusions

Thus this study provides evidence that *ADRM1* plays a role in *ADRM1*-amplified ovarian cancer. The results indicate that amplified *ADRM1* is involved in cell proliferation, migration and survival in ovarian cancer cells, supporting a role as an oncogene and novel therapeutic target for ovarian cancer. Because *ADRM1* is a proteasomal ubiquitin receptor, future work may focus on the susceptibility to proteasome inhibitors in *ADRM1*-amplified ovarian cancer. Additionally, small molecule inhibitors targeting *ADRM1* directly may represent a novel beneficial therapy for 20q13-amplified ovarian cancer, and may act through altering regulation of multiple ovarian cancer targets including *HSP90*s, *MNAT1* and respective cyclins, *ST1A3* and hormone levels, and *HAX1* and apoptosis.

## Figures and Tables

**Table 1:** Difference Gel Electrophoresis (DIGE) and mass spectrometry results in identification of 15 down-regulated and 7 up-regulated proteins in *ADRM1* RNAi-treated OAW42 cells compared to untreated parental and non-targeting siRNA-treated OAW42 cells (not including *ADRM1*).

Av. Ratio	*t*-test	ID	Protein	pI	MW
1.43	0.0019	LAMB1_HUMAN	Laminin subunit beta-1	4.83	205150
1.41	0.00043	LAMC1_HUMAN	Laminin subunit gamma-1	5.01	183191
1.28	0.00016	ITAV_HUMAN	Integrin alpha-V	5.45	117048
1.22	0.00011	IDHC_HUMAN	Isocitrate dehydrogenase (NADP) cytoplasmic	6.53	46915
1.21	0.00017	PLST_HUMAN	Plastin-3	5.41	71279
1.21	0.00024	ST1A3_HUMAN	Sulfotransferase 1A3/1A4	5.68	34288
1.2	2.30E−05	DC1I2_HUMAN	Cytoplasmic dynein 1 intermediate chain 2	5.08	71811
−1.17	0.00012	PURA2_HUMAN	Adenylosuccinate synthetase isozyme 2	6.13	50465
−1.18	0.0053	CAPZB_HUMAN	F-actin-capping protein subunit beta	5.36	31616
−1.2	0.00059	CSDE1_HUMAN	Cold shock domain-containing protein E1	5.88	89684
−1.21	0.00021	KAP0_HUMAN	cAMP-dependent protein kinase type I-alpha regulatory subunit	5.27	43183
−1.25	8.20E−05	SUGT1_HUMAN	Suppressor of G2 allele of SKP1 homolog	5.07	41284
−1.26	0.0083	HS90B_HUMAN	Heat shock protein HSP 90-beta	4.97	83554
−1.31	0.033	HS90A_HUMAN	Heat shock protein HSP 90-alpha	4.94	85006
−1.33	0.031	H90B3_HUMAN	Putative heat shock protein HSP 90-beta-3	4.71	68624
−1.33	0.0038	H90B3_HUMAN	Putative heat shock protein HSP 90-beta-3	4.71	68624
−1.39	0.0098	HS90A_HUMAN	Heat shock protein HSP 90-alpha	4.94	85006
−1.44	0.0024	H90B3_HUMAN	Putative heat shock protein HSP 90-beta-3	4.71	68626
−1.47	0.00026	H90B3_HUMAN	Putative heat shock protein HSP 90-beta-3	4.71	68626
−1.48	1.50E−06	CALU_HUMAN	Calumenin	4.47	37198
−1.5	0.0053	HAX1_HUMAN	HCLS1-associated protein X-1	4.76	31601
−1.59	1.30E−06	MAT1_HUMAN	CDK-activating kinase assembly factor MAT1	5.79	36256
−1.78	4.00E−07	ADRM1_HUMAN	Proteasomal ubiquitin receptor ADRM1	4.96	42412
−2.11	1.40E−09	ADRM1_HUMAN	Proteasomal ubiquitin receptor ADRM1	4.96	42412
